# Austin District Medical Society—Sixth Quarterly Meeting

**Published:** 1889-02

**Authors:** 


### AUSTIN DISTRICT MEDICAL SOCIETY—SIXTH QUARTERLY MEETING.

The sixth quarterly meeting of the Austin District Medical Society will be held in Austin, on Thursday, 21st of March.

So far the following papers have been promised: "Vis Medicatrix Naturæ,—How far physicians should trust to it, etc.," by J. W. Harrison, M. D., of Dripping Springs.

"Mastoid Abscesses," by T. J. Tyner, M. D., Austin.

"Carbolic Acid Poisoning," by W. T. Richmond, M. D., Manor.

There will also be papers on "Pneumonia" and "Hæmaturia," and perhaps other subjects, all of which will be mentioned in the circular of invitation, to be issued on 21st inst.

All reputable physicians accessible to Austin are cordially invited to attend the meetings of this flourishing Society. It now numbers fifty-five of the most prominent medical men in this portion of the State, and has an average attendance upon its meetings of over thirty physicians.

Practical papers and reports of interesting cases always welcome.

